# Does it matter if clinicians recruiting for a trial don't understand what the trial is really about? Qualitative study of surgeons' experiences of participation in a pragmatic multi-centre RCT

**DOI:** 10.1186/1745-6215-8-4

**Published:** 2007-01-27

**Authors:** Sue Ziebland, Katie Featherstone, Claire Snowdon, Karen Barker, Helen Frost, Jeremy Fairbank

**Affiliations:** 1 Department of Primary Health Care, University of Oxford, Oxford, UK; 2 ESRC Centre for Economic and Social Aspects of Genomics (CESAGen), School of Social Sciences, Cardiff University, Cardiff, UK; 3 Centre for Family Research, University of Cambridge, Cambridge, UK; 4 Department of Physiotherapy, Nuffield Orthopaedic Centre, Oxford, UK; 5 Health Sciences Research Institute, University of Warwick, Coventry, UK; 6 Department of Orthopaedic Surgery, Nuffield Orthopaedic Centre, Oxford, UK; 7 Medical Statistics Unit, London School of Hygiene and Tropical Medicine, Keppel Street, London WC1E 7HT, UK

## Abstract

**Background:**

Qualitative methods are increasingly used to study the process of clinical trials and patients understanding of the rationale for trials, randomisation and reasons for taking part or refusing. Patients' understandings are inevitably influenced by the recruiting clinician's understanding of the trial, yet relatively little qualitative work has explored clinicians' perceptions and understandings of trials. This study interviewed surgeons shortly after the multi-centre, pragmatic RCT in which they had participated had been completed.

**Methods:**

We used in-depth interviews with surgeons who participated in the Spine Stabilisation Trial (a pragmatic RCT) to explore their understanding of the trial purpose and how this understanding had influenced their recruitment procedures and interpretation of the results. A purposive sample of eleven participating surgeons was chosen from 8 of the 15 UK trial centres.

**Results:**

Although the surgeons thought that the trial was addressing an important question there was little agreement about what this question was: although it was a trial of 'equivalent' treatments, some thought that it was a trial of surgery, others a trial of rehabilitation and others that it was exploring what to do with patients in whom all other treatment options had been unsuccessful. The surgeons we interviewed were not aware of the rationale for the pragmatic inclusion criteria and nearly all were completely baffled about the meaning of 'equipoise'. Misunderstandings about the entry criteria were an important source of confusion about the results and led to reluctance to apply the results to their own practice.

**Conclusion:**

The study suggests several lessons for the conduct of future multi-centre trials. Recruiting surgeons (and other clinicians) may not be familiar with the rationale for pragmatic designs and may need to be regularly reminded about the purpose during the study. Reassurance may be necessary that a pragmatic design is not considered a design fault. We conclude that it does matter if clinicians do not understand the rationale for the trial if, as we have shown here, their perception of the trial aims and methods adversely affects who they recruit; if their views affect what the patients are told; and if they mistakenly view the results as unscientific, unreliable and ultimately irrelevant to their practice.

## Background

Qualitative research methods are used increasingly to study the process of RCTs. Such studies have predominantly involved assessment of patient participants' experiences of consent, and their understanding of trial design and random allocation [1]. Research addressing these same issues for clinician participants is rare. This is particularly the case for surgeons, in spite of the fact that commentators have suggested that surgical RCTs are particularly problematic. Reviews conducted from the mid 1960s onwards have consistently indicated that RCTs are less common in surgery [2-5]. The quality of surgical RCTs and the standard of their reports are often considered to be low [6-9].

Several authors contend that the culture and mindset of surgeons may be highly influential. Stirrat [10] suggests that surgeons are relatively less involved in RCT participation and draw less upon RCT findings to inform and affect their practice. This may be a residual effect of traditional education [11] and a lack of emphasis on the use of the RCT [12] and epidemiological methods training [13]. In the surgical environment there is a preference for non-randomised studies [5,11] and an informal approach to innovation and research is tolerated [14-16]. Surgeons may be influenced by individual cases with particularly good or bad results. Maddern refers to the impact of "the surgical temperament" which he suggests "does not always lead to well-developed team skills among surgeons" [17] and McCulloch and colleagues argue that traits which are advantageous in a surgeon, namely "comfort with making important decisions quickly with incomplete information", may make it less likely that they will be in a state of equipoise, that is "consciously uncertain which of two treatments is better" [18].

Given these concerns it is surprising that surgeons' reactions to RCTs and their understanding of the methods involved have received so little empirical attention. One study found UK surgical oncologists to be less research oriented than radiation and medical oncologists and more likely to "rely on clinical experience rather than enter patients into a trial" [19]. Surgeons placed less emphasis on association with research, publishing papers, acting as a principal investigator, and having a national or international reputation. They were more likely to feel that hospital-based doctors were given greater rewards for work with patients than for research contributions. Another study of French surgeons, found older surgeons less likely to participate and to use trial results than their younger counterparts [20,21].

These studies suggest some support for the views of the commentators on surgical trials, but further attitudinal and experiential research is needed. It is important to consider how surgeons understand and respond to their involvement in specific trials and to explore how this might affect both recruitment and utilisation of research findings. Qualitative research methods cannot be used to explore cause and effect – for example whether particular clusters of views predict different recruitment rates – but they are ideal for explorative studies that aim to identify perceptions and uncover meanings. This paper reports the views of surgeon participants in the Spine Stabilisation Trial (SST) and explores whether clinicians awareness of the study purpose and rationale is likely to be important.

### The Spine Stabilisation Trial (SST)

This multi-centre MRC-funded RCT compared an intensive functional rehabilitation programme (FRP) with spinal fusion surgery for treatment of chronic low back pain and has recently reported results [22,23]. Spinal fusion for back pain was considered 'the most controversial surgery' in the area of spinal surgery. The SST was a pragmatic rather than an experimental trial, and a particular aim was to assess the interventions in exactly the variable clinical contexts in which they would be used.

The pragmatic design developed from discussions at pre-trial orthopaedic meetings at the Nuffield Orthopaedic Centre, Oxford with contributions from a number of spinal surgeons. The original plan was to conduct an RCT of surgery with a control arm of non-treatment or delayed treatment. It was ultimately decided that an alternative, equivalent treatment should be offered. As trials identifying the efficacy of FRP had already been conducted at Oxford, it was decided to include this as an equivalent treatment. Defining clinical criteria for trial entry was a major problem for the SST designers, even with access to literature reviews, and formal meetings with specialist collaborators. As surgeons across the UK vary greatly in their use of spine stabilisation there was clearly no consensus over the most appropriate patients for this form of surgery. The trial was therefore designed to reflect the variety inherent in clinical practice. The patients who were eligible for the trial were therefore a clinically varied group; aged 18 to 55 with more than a 12 month history of back pain where standard non-operative treatment had failed, about whom the surgeons were uncertain whether spinal fusion or FRP would be the best option.

Recruitment was slow and the numbers enrolled smaller than planned. Fifteen UK centres recruited 349 participants, a third of those originally anticipated. Despite a small sample, secondary power analyses had demonstrated that, with an unexpectedly limited spread of diagnosis (one of the stratification variables for randomisation), the trial was adequately powered. There were sufficient data to analyse and interpret the primary outcomes for the "Chronic Pain Group", but insufficient numbers to explore these outcomes in the "Spondylolisthesis Group" or the "post-laminectomy Group".

## Methods

The purposive sample for this qualitative study was selected to represent different rates of institutional involvement with SST and geographical spread. The recruitment rates for the 15 SST centres were categorised as high, medium and low. Eleven surgeons from eight high, medium and low centres from the South, Midlands and North, were invited to take part in individual, face to face interviews. None refused. Their personal recruitment rates ranged from 0–63 randomised patients and have for this analysis been categorised as high, medium or low.

With informed consent interviews were conducted by a qualitative researcher (DP) with a social science background and experience of interviewing surgeons. Interviews took place during 2004 after the main trial results were presented at an orthopaedic conference but before publication. Qualitative thematic analysis was conducted by two social scientists (SZ, KF) who read and independently coded transcripts and discussed interpretation of the data in a series of meetings and email exchanges with the other authors. Level of agreement was not compared statistically. As is usual in qualitative research differences of interpretation tended to be subtle and were resolved by discussion. Anticipated and emergent themes in the data were analysed using constant comparison and examination of deviant cases [24].

Surgeons were asked their opinion of the SST's research question as well as questions about the purpose and design of the trial; how they became involved; their recruitment procedures including explaining randomisation and uncertainty; their understanding of equipoise and uncertainty in relation to SST; interpretation of SST results, and what might have made the trial easier to conduct. Here we focus on surgeons' understanding of the design and purpose and interpretation of the trial results.

The study was approved by Eastern MREC. Because orthopaedic surgery is a relatively close knit community in the UK we have taken particular care to ensure that the quotations, which illustrate the main themes, are fully anonymised. Therefore when presenting quotes from the surgeons we specify only whether their personal recruitment level was 'high' 'medium' or 'low'.

## Results

### The use of RCT methods

The surgeons expressed positive views of the use of RCTs for examining the effects of medical interventions. Nearly all said that the SST addressed an important question, involved an experienced and respected research team and used the 'gold standard' research method:

*It was well thought out. You know a lot of work was put into it. I mean we had two collaborators meeting; we had a hundred statisticians ferreting about so yes I think the design was good*. Surgeon with low personal recruitment

There were however reservations about the SST design, despite positive views of RCTs in general. For example one surgeon wondered if there are better ways to gather reliable data about surgery in a shorter time frame and suspected that the RCT was used because it was 'politically correct'.

The surgeons were asked what they understood by 'equipoise' and 'uncertainty principle' in relation to the SST. Nearly all were unclear on the meaning of 'equipoise'. Although uncertainty was a familiar term, surgeons often related it to the outcomes of any surgery for an *individual *rather than to the specific comparison of interventions in a trial, here spinal fusion and the FRP (Functional Rehabilitation Programme).

### (Mis) understandings about trial design

It became clear that the surgeons held different views of the purpose and design of the SST. Surgeons were often unsure of the trial aims, were unclear about the nature of the comparison, and expressed concerns about flexible entry criteria.

The purpose of the SST was to compare surgery to an intensive functional rehabilitation programme (FRP) for treatment of chronic low back pain. However, most of the surgeons we interviewed saw it as a trial of surgery, some thought it was a trial of rehabilitation, and others suggested a much broader remit.

I just thought it was a way of sort of trying to work out what's the best treatment for patients, with back problems, that you didn't know what to do with. Surgeon with low personal recruitment

[They] had the great [pause] foresight to actually ask a question which is you know, it's the big question that is in front of us every day when we come to work. Does surgery work? Is it worth doing? You know, that was, what, what, what bigger question can you ask? Surgeon with medium personal recruitment

None of the surgeons interviewed stated that it was designed to compare two 'equivalent' treatments, although this was a key feature of the trial. Many eligible patients had already received extensive physiotherapy and the surgeons described the need to 'dress up', 'talk up' or 'sex up' the FRP arm as something different or new. Some, in contrast, said that they presented FRP as the 'control' or 'conservative arm' in their recruitment 'spiel'.

The SST involved a pragmatic design, comparing interventions as used in clinical practice, rather than in the rigid and artificial circumstances created by explanatory trials. Broad eligibility criteria reflected the fact that surgeons, within and without the trial, vary in their views of which patients might benefit from surgery (several commented on the large difference in the numbers of fusions performed by UK spinal surgeons). The pragmatic design was not understood by the surgeons. Several expressed concern what they perceived as an unfortunate variability in the SST sample and expected that colleagues' perceptions of eligible patients would be different from their own. They talked about psychological and social factors in selecting patients for spinal fusion and the 'art', 'instinct' and 'eye' of the surgeon. Although only consultant-grade specialist surgeons identified candidates for the SST, variations in skill and preferences caused concern. The inclusion of patients with a range of clinical presentations left some feeling that the SST used a flawed design which would render the results unsound or irrelevant to their own practice. One surgeon argued that the broad inclusion criteria loaded the trial against spinal fusion and that, counter to standard trial procedures, they should have been highly selective:

*[If] you want to come out with an answer which says 'spinal fusion works for a selected group of patients', you select the patients very, very carefully for those patients whom you have the least uncertainty for offering the fusion. .... I think that if you actually bias the selection of patients going into the trial to the patients who, in whom the outcome was seen at the beginning to be most uncertain, then you are going to end up with a load of bad apples in the trial who aren't going to do terribly well and I think that's why a lot of surgeons weren't – were fairly diffident about going into it at the beginning*. *Surgeon with medium personal recruitment*

Others also thought the trial was biased against spinal fusion because the 'uncertainty of outcome principle' meant that the most 'promising' patients would have been excluded by their surgeon i.e. they would be given spinal fusion outwith the trial. As one put it '*It's not the test of spinal fusion. It's a test of spinal fusion in a group of patients nobody knows what to do with'*. One consultant suspected that others, like himself, would also take the psychological, social and intellectual profile of potential participants into account when recruiting. Several surgeons felt that these difficulties and problems with recruitment and retention affected the validity of the trial.

These views may have affected the SST. Some surgeons indicated that when recruitment difficulties became public, they had wondered if there was any point in maintaining their recruitment efforts if the trial was destined to 'close early'.

### Making sense of the trial results

The SST allowed recruiting surgeons to exercise clinical judgement. The variation in the rates of fusion surgery amongst surgeons, differences in their perceptions of appropriate surgical candidates and in preferred operative techniques, were all accommodated in the trial design. This inherent flexibility did, however, leave many unsure how to interpret the results. Some of the accounts suggested that there had been considerable discussion within the orthopaedic community about the study and the danger of 'bias' in the design. One surgeon commented: "*We all thought the trial would rather go against spinal fusion because you are recruiting patients with a high degree of uncertainty." Surgeon with medium personal recruitment*

As the SST results had not been published at the time of the interviews, surgeons had not had an opportunity to examine them in detail, but most had received some information about the findings. The principal investigator, Jeremy Fairbank, had presented the findings at a conference and to some colleagues individually. Everyone we interviewed felt they knew the main findings, however, only one surgeon was able to demonstrate a good understanding of the results:

Rehabilitation is more or less as effective as surgery in the treatment of chronic lower back pain in a particular group of patients. That's number one. Number two, recommendations for the future are that people with chronic lower back pain in this situation go and have a rehabilitation programme. If they fail that then it may be appropriate to treat as surgery. We don't know the answer to that. Surgeon with high personal recruitment

Those who accepted that the short-term follow-up showed no real difference between the treatment arms concluded that rehabilitation was a viable and cheaper option. Some of these surgeons were keen to use the results of the study to support their campaigns for better rehabilitation programmes within their Hospital Trusts, although there were also concerns that the results might be misinterpreted or 'misread' as suggesting that spinal fusion should not be funded.

*I suspect that some of the PCTs (primary care trusts) will look at it and say 'Well we should stop spine surgery'. But on the optimistic side, some people may look at it and say 'Well look spine surgery should therefore be used for the physiotherapy failures', which begs the question of 'Why should surgery in that sense be any better?' So like all research you have some questions answered that throws up some more*. Surgeon with low personal recruitment

The results could also be used to persuade patients that surgery might not be the best option, for example, one surgeon said that the results would help him to recommend his patients complete FRP before considering surgery. However, some stressed that the results would not change their practice, because they had always presented the outcomes of spinal fusion as 'very uncertain'.

Concerns about the design and conduct of the SST, largely based on misunderstandings about the nature of the trial, meant that not all participating surgeons accepted the results. A low recruiting surgeon, clearly unconvinced that the trial was adequately powered, suggested that a bigger trial was needed to find a significant difference between the two treatments. Some were disappointed that the trial did not come down firmly in favour of one treatment or expressed disappointment that the trial did not identify which patients would be likely to benefit most from spinal fusion (something which was outside the remit of the SST). A common belief was that the results did not apply to their practice. A surgeon who saw the trial as 'not that valid' described treatment for back pain as 'a personal journey between you and the patient' and suggested that:

[As] long as you have audited your own practice, and you can show that in your own practice that patients who have fusions, in general, do get very good outcomes, then it's perfectly ethical to continue to do. Surgeon with medium personal recruitment

Collaboration with the trial had motivated many of these surgeons to reflect upon and compare aspects of their practice. Some thought it might be more relevant to real patients and practice to pool routine, anonymised, national and international audit data that would enable them to compare and evaluate their practice.

## Discussion

The study adds to the scant literature on clinician's perspectives and understandings of trials. It reports how a small, purposively sampled, group of surgeons who had recently taken part in a specific clinical trial perceived both the trial methods and the results. The study suggests, in line with much of the non-empirical literature, that trials in a surgical context can be particularly challenging. One aspect of this challenge may relate to the views of surgeons themselves and their understandings of the trial design.

The study was funded after the trial was complete; hence all interviews with surgeons took place as soon as possible after the trial closed A qualitative study running concurrently with the trial – especially if it had included observations of recruitment procedures – would likely have yielded additional material, yet might have raised the surgeons' awareness of the trial objectives and rationale. While this might have been beneficial from the SST perspective, the follow-up design identified some quite major misunderstandings that could be averted in future multi-centre trials. We were also able to explore the participants interpretation of the early results and the implications for their practice.

Because of existing controversy on the subject of surgical spinal fusion equipoise was explicitly built into the trial design. Surgeons were asked to recruit patients for whom they personally were uncertain whether spinal fusion or FRP would be the best option. However, the role and importance of equipoise and the 'uncertainty principle' in this situation was not well understood by most of the surgeons. This may in part relate to the fact that they were used to using the term "uncertainty" in a rather different way, relating to the unpredictable nature of any surgical procedure for any patient. This is in contrast with a study of neonatologists where almost all used the concept of equipoise or uncertainty in their interview and seemed to find them useful in trial-related practice [25].

Many of the surgeons were concerned that a patient's eligibility for the trial was dependent on the recruiting surgeon's opinion on their suitability for surgery rather than a clearer set of inclusion and exclusion criteria. Although this is a key feature of pragmatic designs, this was an important issue for the surgeons as it appeared to define the standards of the research. It also undermined the credibility of the results for those who felt that the design of the trial was stacked against spinal fusion from the start, through the inclusion of patients who may be less likely to benefit from surgery. The concerns expressed here echo the pre-trial difficulties in defining appropriate entry criteria for the SST. Whilst the pragmatic element of the design was intended to reflect current practice and variation, this was widely misunderstood. As the surgeons who we interviewed seemed to view the completed trial as explanatory rather than pragmatic, the design appeared to fall short of the highly controlled standards that they expected from experimental research [26].

Confusion among these surgeons about the aims of the trial and the perceived imprecision of the eligibility criteria meant that many were unconvinced that the sample of trial participants were representative of *their *patients. This may in part relate to pre-existing preferences for non-randomised research, as suggested by some commentators, but it has also been suggested that surgeons may be particularly distrustful of the applicability of trial results for individual patients within their own practice. Stirrat suggests that in order to justify carrying out invasive procedures, "the surgeon [has] to travel further along the road of self-belief than his physician colleagues." He argues that as a result they may be less likely to be reflexive than other physicians, and are used to viewing patients as individuals rather than as part of a population or community, factors which may reduce faith in the value of RCTs [10]. Buchwald claims that some methods that are fundamental to the conduct of trials, can be alien concepts for many surgeons [11].

## Conclusion

The SST trialists performed according to current good practice guidelines, for example holding collaborators meetings and producing a video to explain the rationale for the trial. However, by the end of the trial some of the key characteristics of the trial were not recalled by participating surgeons. Recruitment took place between 1996 and 2002, which is a long time to hold the interest of treating clinicians. We do not suggest that the SST was a poorly conducted trial, rather that this opportunity to explore surgeons' (mis)understandings of the trial has allowed us to identify issues with broad application for clinical trials.

This qualitative study suggests several lessons for the design and conduct of future multi-centre trials. Knowledge of the principles of trial design and the differences between pragmatic and explanatory trials amongst clinicians at any level should not be taken for granted. In the SST misunderstandings about the entry criteria were an important source of confusion about the applicability of the results. Trialists should be clearer about which patients can be included and why, and should explain to recruiting clinicians that trial statisticians are able to control for patient variation. This point – and others – will likely need to be reinforced during the conduct of a trial that lasts for several years. It is the responsibility of trialists to ensure that training on the specific aims of the trial and rationale for the design should be attended by all recruiting clinicians before they join the trial. We suggest that this should be regularly reinforced during the course of the study, especially in trials that do not use a straightforward placebo controlled experimental design.

Investigators keen to nurture and retain potential collaborators may be inclined to waive attendance at training for senior colleagues, but motivation to participate in prestigious trials (such as the SST) remains relatively high; the majority of these surgeons recognised the benefits of being attached to a high profile MRC study. We can also assume that the surgical community would not be the only sector to benefit if awareness of appropriate trial design were to be developed among clinicians.

The clinical question the trial is asking is most likely to be understood and remembered if it is kept simple and reinforced by a simple strap line on all trial literature. With the benefit of hindsight the title 'Spine Stabilisation Trial' does not suggest a comparison of two equivalent treatments – one surgical, one rehabilitation. Thus, it is perhaps not so surprising that a few years later some participating clinicians were often unable to recall the involvement of the rehabilitation arm and were in a poor position to interpret the significance of the results for their own practice. A similar issue was raised in relation to the UK Collaborative Neonatal ECMO Trial in which people often referred to the trial and the experimental intervention (ECMO) interchangeably [27].

In conclusion, does it matter if participating surgeons didn't understand what the trial was about? We would argue that it does if, as we have shown here, their perception of the trial aims and methods adversely affects who they recruit; if their views affect what the patients are told; and if they mistakenly view the results are unscientific, unreliable and ultimately irrelevant to their practice.

## Competing interests statement

JF and HF were involved in the design of the SST and JF, HF and KB in its implementation.

## Authors' contributions

JF, HF, KB and SZ were grant holders and designed the study with advice from KF. SZ led this analysis with considerable input from KF and CS, and drafted the paper. All authors contributed to subsequent drafts and read and approved the final version.

## Note

Sue Ziebland is guarantor for this paper.

Ethical approval was granted by Eastern MREC.

We are grateful to the MRC for funding this study, to all the surgeons who took part in the interviews and to Dorothy Pryce for her contribution to the fieldwork
